# Real-Time Emotion Recognition for Improving the Teaching–Learning Process: A Scoping Review

**DOI:** 10.3390/jimaging10120313

**Published:** 2024-12-09

**Authors:** Cèlia Llurba, Ramon Palau

**Affiliations:** Department of Pedagogy, University Rovira i Virgili, 43007 Tarragona, Spain; ramon.palau@urv.cat

**Keywords:** educational technology, teaching skills, learning processes, emotional behavior

## Abstract

Emotion recognition (ER) is gaining popularity in various fields, including education. The benefits of ER in the classroom for educational purposes, such as improving students’ academic performance, are gradually becoming known. Thus, real-time ER is proving to be a valuable tool for teachers as well as for students. However, its feasibility in educational settings requires further exploration. This review offers learning experiences based on real-time ER with students to explore their potential in learning and in improving their academic achievement. The purpose is to present evidence of good implementation and suggestions for their successful application. The content analysis finds that most of the practices lead to significant improvements in terms of educational purposes. Nevertheless, the analysis identifies problems that might block the implementation of these practices in the classroom and in education; among the obstacles identified are the absence of privacy of the students and the support needs of the students. We conclude that artificial intelligence (AI) and ER are potential tools to approach the needs in ordinary classrooms, although reliable automatic recognition is still a challenge for researchers to achieve the best ER feature in real time, given the high input data variability.

## 1. Introduction

Emotions are of vital importance in society and have a large impact on the way we express ourselves on a daily basis. The rapid development of sensors and information technology has made it possible for machines to recognize and analyze human emotions [1]. Thus, the most significant components of human mental state are introduced into machines so that they can automatically detect the emotions expressed by a human being through their facial features [2].

The methods of emotion assessment presented in the literature can be classified into two groups according to the techniques used for ER, by answering questionnaires, or by automatic assessment techniques based on measurements of various parameters of the human body [3]. ER is the identification and interpretation of emotions from multiple sources, like speech, facial expressions, tone of voice, and body language. In addition, several methods are used simultaneously to increase the reliability of the results obtained.

In recent years, the advances in machine learning (ML) and computer vision have made it possible to develop sophisticated algorithms that are capable of analyzing and interpreting ER in different ways [4]. In other words, it is an automated detection system that is used to categorize the values of variables to their subsequent classes, and that involves decision making using ML or deep learning (DL) techniques [5].

Thus, ML contributes to a more inclusive and accessible education by transforming the learning process and providing new tools to education to improve student performance and engagement. Moreover, real-time ER based on facial expression is an emerging field of research that is increasingly being proposed in education [6].

Similarly, human–computer interaction is becoming increasingly important as we rely more and more on digital systems, gadgets, and apps in our daily lives [7]. Therefore, nowadays, the union of human psychology with new technologies, such as these intelligence systems, allows us to have a better interaction, since the availability of data on human emotions is a promising advance [8]. Computer vision techniques using AI algorithms can detect and recognize facial emotions, considering the emotional theories and how to assess them [9], and it is not only interesting in terms of understanding these emotions, but also serves as a great help to improve various technological applications in different fields, such as health, human–machine vision, and education, among others.

Leveraging AI for ER profoundly benefits people and humanity [10]. Moreover, integrating ER into AI aligns with the growing emphasis on human-centered technology, fostering a more nuanced and adaptive relationship between people and the digital realm [11]. The emergence and advancement of automatic facial expression recognition systems can greatly increase the amount of data processed. In addition, if the ER is in real time, the application can boost society [2].

Also based on [12], emotion-rich experiences can lead to a deeper and more lasting internalization of information, which, in the long term, can influence the teaching–learning process. Like this, monitoring students’ emotions can contribute to fostering a positive classroom climate [13] and creating healthier and more equitable educational communities by promoting socioemotional well-being among students [14]. Thus, focusing on the feeling’s associated perception and processing of new information acquired in class may constitute an essential component of the knowledge and skills acquired for students [15].

Further, emotions play an essential part in our daily life, and it is enough to recognize students’ emotions in class related to learning [16], since they control the student’s attention [17], affecting their motivation to learn and influencing their self-regulated learning [18]; also according to [19], auto regulated learning and motivation influence the effects of emotions on educational achievement. Moreover, emotions have a significant impact on motivation, the ability to relate to others, and the ability to regulate stress [20]. Among other research [21,22], and building on [23] the theory of the control value of achievement emotions, the definition of emotion can help educators distinguish emotions from other affective phenomena, such as moods and stress, terms closely related to emotions, although they are not interchangeable and can be differentiated [24].

Emotions, defined as a dynamic bodily disposition that is at the basis of actions and underlies all human action [25,26], have been addressed by many authors in the fields of philosophy and psychology. The author [27] proposed that emotions have a biological basis and that facial expressions of emotions have an evolutionary origin. Then, [28] added that emotions are perceptions of a specific body that change in response to emotional stimuli. Based on this, the authors of [29] worked with James to develop the theory that emotions are the result of the perception of bodily responses that occur in emotional situations; still, [30] suggested that unconscious conflicts can influence emotions, and [31] proposed that emotions are the result of the cognitive evaluations related to our personal goals and values.

Subsequently, Ekman and Friesen focused on universal emotions and facial expressions [32], stating that there are six basic universal emotions that are recognized in different cultures and can be divided into six different categories: happiness, sadness, surprise, disgust, anger, and fear, besides the neutral emotion [33].

Teachers observe students’ emotions and facial behaviors during class, thereby obtaining feedback. Student behaviors are diverse and complex, making it difficult to obtain a holistic understanding of each student’s performance. In addition, teachers teach one at a time, which makes it even more difficult to obtain a comprehensive and in-depth assessment of each student’s performance in the classroom [34]. Student motivation and emotions are claimed to contribute to student engagement and learning [35]; however, translating research on motivation and emotions into educational practice and policy has so far been difficult. Although a main goal is to improve the teaching–learning process, knowing students’ emotions is very significant, and it is also necessary to analyze classroom behavior to help the teacher raise the quality of teaching and learning; thus, a posteriori analytic methodologies may help teachers to reflect on the quality of teaching.

Moreover, modern teaching has experienced enormous progress thanks to the introduction of a wide range of equipment and advanced technologies in the teaching process. Facial expression recognition algorithms are being increasingly used as the dominant emotion detection technological approach, due to scientific developments in computer vision using DL analysis [36]. Thus, as technology progresses, new models can be expected to produce increasingly efficient results. For example, neural networks have greatly increased the performance of models and improved accuracy [37]. Methods are being developed to predict facial expressions, encode facial expressions, and mine these characteristics so that machines can better predict them, with the aim of creating a dataset to detect facial emotion [37]. The success lies in achieving a better performance, so there are different techniques and modalities, of which the most outstanding are explained in the present review.

## 2. Materials and Methods

The present article is a scoping review of the literature on real-time ER based on experiences with students during a lesson. The review is based on the Prisma statement [38] in the selection of information sources, search strategy, steps, and the procedure of the selection process, and it used the Mendeley program [39] for the management of bibliographic references.

### 2.1. Scope and Research Questions

The scope of this review is narrow, which includes real-time ER on not being used in all types of learning scenarios and experiences but being limited to those designed for education-related centers. The research does not include studies reporting on instructional design in e-learning studies, but does include analysis of face-to-face classes for possible use in smart classrooms.

The research questions are the following (Table 1):

### 2.2. Eligibility Criteria

The inclusion criteria were (1) studies focusing on real-time ER on for educational purposes for students, independently of the methodology and whether the results are positive or negative; (2) studies that include at least one student but specially a group of students; (3) studies providing detailed information on the instructional design; (4) studies published and available in full-text form; (5) studies written in English, and (6) publications after 2018.

The exclusion criteria were (1) studies reporting experiences with non-real-time ER; (2) studies reporting learning experiences different for educational purposes; (3) studies involved in e-learning or virtual classes; and (4) systematic reviews.

Eligibility criteria are synthetized in Table 2.

### 2.3. Sources and Search Strategy

The results were obtained through two multidisciplinary databases, Scopus and Web of Science (WoS), and also hand-searching. We filtered the results to selected papers published after 2018 in the English language, in the following subject areas: Computer Science and Social Sciences. All searches were performed during the last six years, from 2018 to March 2024. The total number of articles acquired was 58 (18 from Scopus, 28 from WoS, and 12 from hand-searching).

The most useful results on the Scopus database were achieved by searching within the article title, abstract, keywords, and documents related to “REAL TIME EMOTION RECOGNITION” AND documents related to “EDUCATION” OR “CLASSROOM” OR “ACADEMIC PERFORMANCE”. The total number of articles obtained from Scopus was 18. Of these 18 documents found on Scopus, five articles were not related to education, leaving **13** articles remaining.

On the other hand, the most useful results on the WoS database were obtained searching within TS = (REAL TIME EMOTION RECOGNITION* AND CLASSROOM* and EDUCATION*). The total number of articles obtained from WoS was 28. Of these 28 documents found on WoS, 13 articles were neither related to real-time ER nor education, leaving **15** articles remaining. In addition to searching reputable databases, the screening of hand-searched articles was added. These articles (12) reviewed for the same content, similar to the full-text review, left **9** hand-searched articles remaining.

Finally, after reading these **37** papers, **22** were found to fulfill the requirements for inclusion.

### 2.4. Selection of Studies

**Figure 1:**
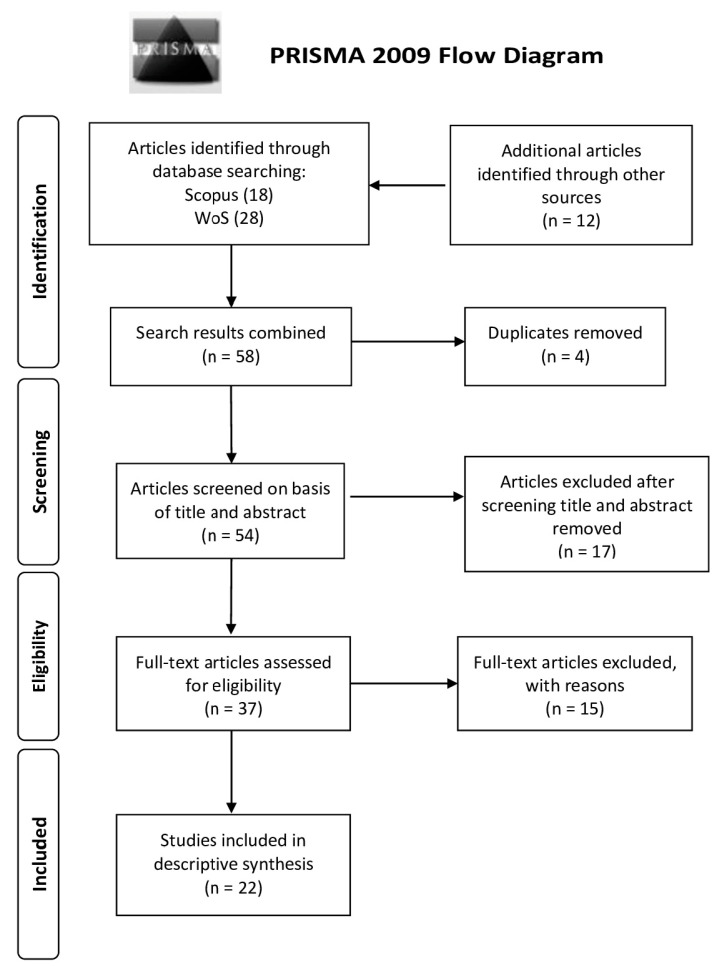
Study selection process flow diagram.

Eligible articles filtered by screening were on the basis of their titles and abstracts. A total of 37 articles were filtered, and full texts were collected; finally, a dataset of 22 papers was selected for the scoping analysis. The number of studies included and excluded is summarized in Figure 1.

## 3. Results

The database search (18) in combination with hand searching (4) resulted in a total of **22** articles that met the inclusion criteria for review. 

**Figure 2:**
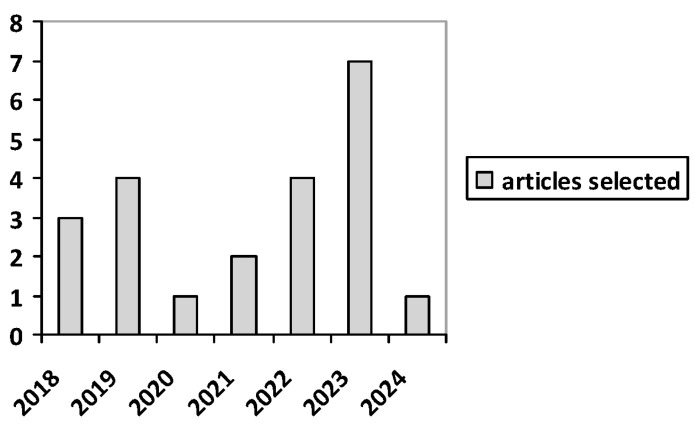
Number of publications by year of the 22 articles included in the scoping review.

Of these 22 articles included in the scoping review, the number of publications by year is shown below in the Figure 2. 

### 3.1. Parts of a Real-Time ER

An ER system is generally divided into different steps, starting with face detection until the information is available, as shown in Table 3 and according to [40]. It first detects the face, goes through several steps as data processing and analysis, and ends with the layout of the information.

### 3.2. Experiences of Real-Time ER

In the literature, there are different ways to collect data, so regarding first research question about patterns used for ER, it can be said that [41] collected data using a system with a camera that captures real-time students’ images in the classroom, maybe a high definition camera or set of cameras connected to a desktop computer used to monitor students continuously during the lesson.

When analyzing a facial expression image, the quality of the images is very relevant to recognize the emotion of a student, since real-time facial recognition depends on them [42]. Higher-resolution cameras with the ability to take sharper images will help improve detection, and video recording should be substituted to analyze the effect of time in the study and observe how emotion changes in real time [43]; also to obtain a more accurate result, a number of images taken over time need to be analyzed during the learning process [44].

In addition, authors such as [45] comment that exploring the possibility of reducing the number of layers to obtain a simplified ML architecture of the hybrid model, or using a random set of patches as the image representation, can further improve training time.

For that reason, [34] says it would be more useful if student behavior statistics could serve to provide teachers with a reminder to readjust their teaching style in real time. For instance, if the behavior analysis system in class counts that a high percentage of the students are sad, teachers are able to look at the live statistics screen and know that so many students are not listening and may adjust their teaching strategy by either asking the right questions or repeating the lecture in another way. This will support students to become fluent in the content and enhance the teaching effect, and consequently in turn, academic performance will be improved.

In this way, [34] collected data analysis using integrated magnetic resonance imaging appliances analyzing and displaying in real time to optimize classroom teaching. Other authors [46] included the design of a model that can accurately predict the students’ engagement state by analyzing the direction of their gaze, as well as the state of their eyes and their emotions, having the potentially to positively impact education and society by fostering customized learning experiences and promoting to the development of innovative educational technologies [46]. In turn, [47] proposed a model consisting of the recognition and fusion of multimodal information on head posture, as well as the facial expression analysis and classroom interaction by using multimodal fusion technology to analyze in an intelligent way the students’ interest in learning, in order to comprehensively analyze students’ interest in a teaching scenario; in order to test the validity and viability of the model, full-time graduate students participated in the experiments in real classroom environments. Another pattern used for ER is the Super Star interactive platform, which was also used to collect interactive feedback from students in the classroom; it is a mobile application used to provide a flexible and dynamic learning environment and enhances students’ learning competence.

Besides this, an image is captured with the laptop camera, and the system captures a face in the image [48]. In case there is no face, the system will keep capturing frames until a face is identified. On the other hand, [49] designed a real-time ER system through classroom activity, where students are provided with a questionnaire, the result of which will be applied to examine whether or not the use of the system can decrease the students’ stress. In fact, it shows that the use of the system can detect the mood of the students in advance so that the teacher can minimize the students’ stress. It must be said that [50] followed the line of this research.

Following the ER patterns, a real-time feedback system was proposed by [51] that was based on a matrix camera or even several cameras to collect the facial expressions of the students and judge the teaching impact. As the range was adjusted according to the focal length and resolution of the camera, seven was the number of students whose emotions could be identified simultaneously in one frame.

Incidentally, in the experiment by [52] consisting of 40 participants, they were asked to solve eight visual programming tasks using a Lego Mindstorms EV3 educational robot, while at the same time the participants’ faces were recorded with a tablet placed in front of the computer. It should be noted that the timer of the tablet and the computer were synchronized.

Regarding the second research question about technology used for real-time ER in classrooms, several works can also be mentioned, for example, the experiment by [52], which was based on the development and use of ML models, obtaining real-time facial ER from the images of students’ faces in the classroom environment.

Alternatively, a face recognition algorithm was introduced, relying on a students’ class video [53]. However, one of the main aspects of the project by [4] is the application, which meets the cross-platform requirement, being suitable for most operating systems with the use of a computer; the thread of the model is to capture data with a microphone and a camera sensor connected to the computer that constantly retrieves data from the video and audio sources. The application does not process the missing modality data if there is no video or audio signal. For example, if a face is not detected, the audio signal is introduced to the system and the TFusion module predicts it using a single mode.

By comparison, a speech ER framework reliable and efficient enough to work in real-time environments [54] aims to provide. In contrast, speech ER can be accomplished using both linguistic and paralinguistic aspects of speech; this specific work focuses on the paralinguistic aspects, using non-lexical attributes of speech such as pitch, intensity, and mel-frequency cepstrum coefficients (which is a representation of a short-term power spectrum of a sound) to train supervised ML models for ER.

Meanwhile, new facial features for each emotion are initially detected using a simple and powerful feature descriptor known as a histogram of oriented gradients for face recognition, from which automatic ER is then performed by training a convolutional neural network (CNN) that takes real-time input from a camera deployed in the classroom [2]. The system is able to identify students’ facial expressions with the help of the facial expressions used to train the system, which are continuously stored in a database.

On the other hand, a system to obtain the characteristics of students’ facial expression in business English class is presented by [55], providing an ER model that consists of modules of emotion mechanism, signal acquisition, analysis, recognition, emotion understanding, emotion expression, and wearable equipment.

Additionally, [2] developed an automatically controlled, real-time ER system that incorporates new prominent facial features for classroom assessment using a DL model. The sample group consisted of 100 students.

Conversely, an analysis was performed using a combination of prosodic and spectral features, and the classification was conducted by using algorithms such as Gaussian naïve Bayes, random forest, k-nearest neighbors, support vector machine, and multilayer perceptron [54]. Several pattern recognition algorithms can be used for classification. In this study, the Ryerson audio-visual database of emotional speech and song (RAVDESS) was used. The data were read using Python’s Librosa library, and features were extracted with the help of Librosa and Parselmouth.

By contrast, facial detection was performed by the Viola–Jones algorithm and was deployed using OpenCV for the proposed system [48]. At the same time, [53] enhanced the hybrid face detection model that is based on a conventional model and suggests the neural network algorithm of learner’s expression recognition focusing on a visual transformer.

Besides the real-time intervention system based on the work of [16], they designed an emotional contagion model for the classroom scene to track the source of negative emotional contagion with a DL-based visual ER system for all students in the classroom; they used an edge-computing-based service to minimize response time to achieve multi-person ER and interact in real time.

The method proposed by [56] uses ML involving a real-time ER system; to be more precise, a CNN is used to identify the learner’s emotion in order to obtain more accurate features. Specifically, the MATLAB 9.13 production software environment is used to build the proposed system. Thus, the learner’s emotion can be efficiently identified using Wiener filtering to remove unwanted noise, which once removed is subjected to a segmentation process that provides a high degree of accuracy in the result obtained from the preprocessed image; that is, starting from an image as input for ER, subjected to a variety of treatment methods that make it possible to segment it using a CNN for the extraction of its features.

Another ER modality is the combination through a fusion network to create a multimodal feature vector that is introduced into a layer of classification for such identification. That research proposes a new attention-based approach for multimodal ER, i.e., a method for detecting emotions by fusing and analyzing data from several modalities from which a complete picture of an emotional state of a person can be acquired [57].

Furthermore, [6] propose the implementation of facial expression feature extraction with an end-to-end fusion network based on attention modules to detect different facial emotions with extremely high accuracy.

Rather, from a teacher’s point of view, [34] proposes an integrated appliance plus an AI application solution for classroom behavior analysis to adapt to the development of modern teaching, support teachers in classroom teaching, and enhance teaching quality, while a lightweight model analyzes students’ classroom behavior through ER and statistical analysis with high accuracy and efficiency.

Along the same lines, illustrated in [41] is the YOLO (You Only Look Once) (v5)-based action, emotion, and face behavior detection, which is a fast, precise, and easy-to-use platform system using AI of DL algorithms for automatic assessment of classroom attention; furthermore, it can accurately determine the attention levels of the students (tested on group of seven students) and act as an assistant in decision making, offering strategic information to teachers in real time and offline through the detection of behavioral statistics, emotions, attendance, and progress of students. Finally, the evaluation of the system is shown. Analyzed images with the trained model are used to provide reports for teachers on student actions (these can be reports for individual students and for the class as a whole), attention, and emotions, which are classified as high and low attention.

By comparison, there is a focused study on the detection of commitment with an approach based on computer vision [46]. As discussed, facial expression classification is implemented on 10 undergraduate students, using CNN architecture, a multilayer neural network used for DL algorithms to directly recognize visual patterns by processing the data. In this case, it was trained with MobileNet V2 architecture using FER data for the extraction of emotional features from facial images, which was a multimodal method based on ER and gaze direction with head pose estimation and eye state. This facial ER system includes image and video acquisition, preprocessing, feature extraction, and emotion classification. The faces collected by the webcam are subjected to a pre-processing stage. The main libraries used for this experiment are Tensorflow (backend of the system), Keras (activation function, optimizers, and layers), Numpy, OpenCV (image pre-processing stage), Matplotlib (plotting), and Scikitlearn (for generating the confusion matrix).

Interestingly, such a system [2] is able to identify students’ facial expressions with the help of the facial expressions used to train the system, which are continuously stored in a database. Usually, the programming language is Python, and thanks to the PyAudio library, the audio signal is collected and processed.

Further, high performance in ER tasks is achieved by using bimodal ER supported by the TFusion module, in which innovative network architecture was proposed that provides separate audio and video branches, as in the work [4].

Nevertheless, [58] proposes an ER method that relies on analyzing and detecting students’ real-time sentiment called NAGNet. This network model is composed of Res2Net, non-local attention, and generalized mean pooling, which can fuse the global information of expression features to perform fine-grained sentiment analysis. In addition, training and experiments with the large-scale emotion dataset facial ER (FER+) were conducted, where the images were re-labeled into one of eight emotion types: happiness, sadness, surprise, anger, fear, disgust, contempt, and neutral.

Differently, [59] offers a practical approach for classroom evaluation and improvement of teaching methods in order to achieve intelligent education, as well as providing a DL-based intelligent recognition technology for facial expressions. This model for facial expression recognition of the students in the classroom approaches the problems of instability in the process of such recognition, high parameter redundancy in traditional CNN, long training time, and slow convergence prone to over-fitting, proposing a hybrid attention mechanism in the deep neural network model before feature fusion to extract features with better representational ability, improving the prediction performance of deep neural networks and improving the interpretability of the model, modifying the block convolutional attention module with direct access connections by increasing the network depth of the attention module, and obtaining an improved hybrid attention mechanism that allows it to learn the important information between spatial regions and feature channels efficiently.

In reference to the last research query, the question number 3, and in relation to the literature researched, not much information has been found; there is still a long way to go for implementing the theory into schools’ practice. However, regarding how teachers obtain ER information, it can be said that the results on [cui54] show that the test ER model monitors each student’s emotional states in real time during business English lessons; upon detecting frustration or boredom, the ML will timely switch to the content that is easier to learn or more interesting to the learner, keeping the student active in learning.

It can also point out that time and effort spent on tasks can be minimized with a well-designed interface [7], increasing the teacher’s efficiency. Therefore, thanks to the interface [41], real-time detection of these activities allows the teacher to be aware of the students’ attention and thus adjust the lesson to attract their interest.

### 3.3. Ethical and Privacy Issues

There are learners who are opposed to sharing information on privacy grounds and feel that if their privacy is compromised, it may have a negative impact on their learning [60]. For this reason, issues of learner privacy need to be addressed when we engage in public knowledge sharing in a learning environment. Although some researchers have already discussed the moral tensions and ethical dilemmas in privacy-related issues [61], there are still questions related to the boundaries of privacy and public knowledge sharing that remain to be resolved.

A secure learning environment is protected by ensuring the privacy of learners [62]. In these learning environments, students can be identified by numbers rather than being represented by name; thus, privacy issues related to society and psychology are solved by focusing on learners’ products, not their names. In addition, educational institutions must provide security to protect students’ personal information [63]. As teachers may inform students of the benefits of openly sharing comments, ideas, and critiques in learning environments, when they are in class and registered by an ER, teachers should inform students of the benefits they will receive and the contributions they will make by sharing their work publicly, as well as options for keeping their identity private when they feel uncomfortable sharing their data publicly [60]. When students recognize the value of knowledge, they contribute more in a learning environment and are less likely to resist sharing it publicly [62].

It must be said that Europe’s digital future is being shaped. The use of AI in the EU will be regulated by the AI Act, the first-ever legal framework on AI, which will address the risks of AI and puts Europe in a world-leading position in this regard [64].

Meanwhile, the research protocol must be submitted for consideration, comment, guidance, and approval by the relevant research ethics committee before initiating the study [65].

## 4. Discussion

In recent years, the field of ER has developed several methods based on simple or multimodal approaches to effectively identify students’ emotional states, thanks to the interest of numerous researchers. The ER of students is of great relevance, especially in achieving an improvement in learning processes.

Thus, in this section, and based on the study presented in the previous section, the best approaches are extracted from the different references. The research questions will be addressed and the findings discussed.

On the first question, the patterns used for ER, it can be said that mainly the authors use a camera or several cameras strategically placed in the classroom to focus on the maximum number of students attending the lesson. The camera can be a professional camera, matrix camera, students’ tablet, or even a webcam connected to the computer where data are transferred, which, depending on the authors, will be a set of images or videos. In this way, sensors used for ER can collect signals from different dimensions and achieve emotional analysis using some algorithms. Furthermore, different sensors have different applications in ER. Hence, as previously seen, ER based on visual sensors is one of the most common ER methods within the investigated literature, being characterized by simplicity of data collection and low cost.

Although in most of the selected articles, the detection of emotions is by images or videos that are processed and customized, in other papers, it is seen that instead of detecting the face, the ER is done through the audio; as is stated by [66], human speech contains abundant information that can be used for ER, or with students’ speech, even removing the noise for a greater accuracy.

As follows, different techniques are shown concerning the analysis of selected real-time ER systems, starting from physiological signals (gaze, skin temperature, electrocardiogram, …) as well as psychological signals (speech, gestures, position, facial recognition, …).

It should be noted that according to [67], the analysis of nonverbal communication could be combined with the analysis of verbal communication to take a holistic view of communication patterns and develop an integrated framework for the analysis of communication factors that impact on individual well-being.

Regarding the second question, the technology design used for real-time ER in classrooms mentioned in the chosen articles is based on different options, such as being guided by gaze direction, eye state, and emotion analysis; a new attention-based approach for multimodal ER; with the technology of an end-to-end fusion network based on attention modules; the YOLO platform based on using AI DL algorithms; data collected through integrated magnetic resonance imaging devices; a 3D learning interest model; bimodal ER based on the TFusion module; and using CNN for DL algorithms such as MobilNet CNN, being the most common with ML techniques. In sum, the ability of DL models to automatically extract and classify features is gaining popularity and has increased the use of CNN models. Methods based on neural networks and facial features have shown good performance in recognizing the emotions of students and are highly valuable in the education field [68]. In addition, [40] modeled an optimal system for real-time facial ER that usually consists of three basic steps: facial emotion feature extraction; facial preprocessing; and emotion classification.

In reference to the third question, on how teachers obtain the information, it may be said that the teacher has the information when the system displays the rating and provides them with real-time data, basically as spreadsheet files.

Other authors such as [69] propose a technological tool with the aim of providing information to both teachers and students, helping them to make decisions in a simple and rational way by encouraging personalized learning.

Collected emotions are shown to teachers in an intuitive and simple way by means of colors, which are directly related to the affective feeling. The development of this tool includes a collection of information from students and environmental factors through various devices, showing teachers the information provided so that they can know the emotions of students and those environmental factors that are positively and/or negatively affecting the teaching–learning process. It also suggests actions for the teacher to implement in the classroom and distinguishes between the different types of activities, including an expository class or an individual and group activity. Furthermore, the instructions suggested by the tool are adapted according to variables such as the group, the teacher, or the time of day. At the same time, the tool is trained according to the data collected, allowing the instructions and activities to be adjusted to the needs of the class.

There is another learning analytics tool designed to help teachers called Course Signals [70], which consists of a predictive model to detect students’ risk of failure, as well as a dashboard that uses the traffic light analogy to visualize that risk of failure (green: no risk, yellow: moderate risk, red: high risk). The predictive model is based on a wide range of variables, such as learning management system activity, demographics, and prior academic performance. The use of this predictive model acts as a scalable solution to provide early and timely personalized feedback to students.

Detecting students’ emotions is crucial to promote effective learning because of the inextricable links between cognition and emotions [71], so identifying students’ real-time emotions during the learning process is essential and fundamental to obtain valuable information [72]. In fact, [73] has examined the relationship between emotional factors and student performance, concluding that students with good emotional health are more likely to succeed. At the same time, positive emotions can increase students’ interest and motivation for learning. However, long-term emotional monitoring can put pressure on students, which can affect their participation and willingness to cooperate [68].

These concerns require careful consideration of participants’ welfare and privacy in the application of this technology, due to there being a notable lack of consensus on the issues of safety, ethics, and privacy in this context, further complicating the implementation of ER technology in education [68].

## 5. Conclusions

ER is crucial in multiple fields, including education. ER technology based on intelligent devices and models can support the design and implementation of educational intervention measures.

The present study provides an overview of the current state of the recently published literature on the educational activities of students’ ER while attending class. Educators can better understand students’ psychological states, which guide the way they teach and help shape individualized plans such as support needs, by collecting students’ signals via smart devices and performing real-time analysis with ER models. At the same time, it explores the potential role of emotions in education, focusing on didactic purposes, especially in the improvement of teaching–learning processes.

The paper summarizes the most relevant empirical findings of 22 articles highlighting the main results and suggestions. It shows different techniques of decomposition ER systems for both physiological signals (gaze, skin temperature, and electrocardiogram) and psychological signals (speech, gestures, position, facial recognition). These recent works mainly focus on architecture and deep neural networks, hybrid classifiers, and fusion methods for the ER system.

Considering that a teacher’s job is not easy, as they must take interest in each student and seek for their opinion, it could be said that the application of educational systems adopting facial ER is becoming more and more popular in recent years, due to researchers focusing their attention on effective emotional detection in learning and effective learning behavior to improve the impact and quality of teaching in real time.

Having introduced and described the different approaches to facial ER through the synthesized study of the various existing approaches in this paper, and as mentioned above, real-time education with ER has significant potential for improving inclusive education, such as improvements in student learning, engagement, participation, and social interaction. In addition, the analysis shows that the fusion of features and data helps to improve students’ academic performance. Consequently, ER will undoubtedly enhance the teacher’s ability to perform personalized synthesis and select appropriate pedagogical methods. As a result, ER systems will assist teachers to interact appropriately and accurately in order to foster engaged academic learning.

Nevertheless, ER technology to address emotional changes in education is on the way to further improvement and refinement. Some criteria should be taken into account in the development of the studies, such as specificity and reproducibility, and it would be interesting to further explore obtaining multimodal data that combine multiple sources of information, whether by speech, body movement, facial expression, or eye status, thus obtaining much more reliable ER methods. However, conventional ER methods present some problems such as the absence of student privacy, highlighting the lack of information accessibility due to ethical and privacy issues.

## Figures and Tables

**Table 1:** Research questions (RQ).

RQ	
1	Which patterns of ER are used?
2	Which technology is used for real-time ER in classrooms?
3	How is the technological design in which teachers obtain information?

**Table 2:** Inclusion and exclusion criteria for title–abstract screening.

Inclusion Criteria	Exclusion Criteria
Real-time ER	Non real-time ER
Educational purposes	Non-educational purposes
Face-to-face lessons	E-learning/virtual lessons
Empirical studies	Systematic review
Published after 2018	Published before 2018
Published articles in full-text form	Unpublished articles
English articles	Non-English articles

**Table 3:** Main parts of the real-time ER system.

Parts of an ER System	Definition
Face detection	Algorithm capable of accurately detecting the detection of the presence and facial position in video or images sequences in real time
Pre-processing	Normalizes images, eliminates noise, and enhances contrast to increase the accuracy of feature extraction in real time
Data processing	Can handle the video or image stream and process the extracted features and the results of the classification in real time
Feature extraction	Algorithm which can extract relevant facial features in real time (facial landmarks’ shape or position, facial textures, facial muscle movement)
Training data	Train the classification model through a wide and rich dataset of facial expression annotations
Classification model	ML model such as a deep neural network or vector machine suppor that can learn from the extracted features to categorize various facial expressions in real time
Output and feedback	Real-time system that displays the results and provides the user with information

## Data Availability

There is no research data.
